# WWTR1 (WW domain containing transcription regulator 1)

**DOI:** 10.4267/2042/54169

**Published:** 2014-11-01

**Authors:** Yulei Zhao, Xiaolong Yang

**Affiliations:** Department of Pathology and Molecular Medicine, Queen’s University, Kingston, ON, Canada

**Keywords:** Oncogene, cell differentiation, transcriptional coactivator

## Abstract

WWTR1 (also called TAZ in publications. Therefore, TAZ is used in the following description) is a WW domaing-containing transcriptional coactivator, which was first identified as a 14-3-3 binding protein.

TAZ is the downstream component in the Hippo pathway, and also has been found to interact with different pathways, such as Wnt, TGFbeta, etc. TAZ is involved in mesenchymal stem cell differentiation as well as tumorigenesis.

High level of TAZ has been found in different cancers, such as breast cancer, colon cancer, lung cancer, etc.

## Identity

**Other names:** TAZ

**HGNC (Hugo):** WWTR1

**Location:** 3q25.1

**Local order** TM4SF4-WWTR1-COMMD2-ANKUB1.

## DNA/RNA

### Description

TAZ maps to NC_000003.12, in the region between 149235022 to 149454501 and spans 220 kilobases. TAZ has 7 exons, ranging in size from 112 bp to 3754 bp.

### Transcription

The mRNA transcript spans 5135 bp with 1202 bp open reading frame.

**Figure:**
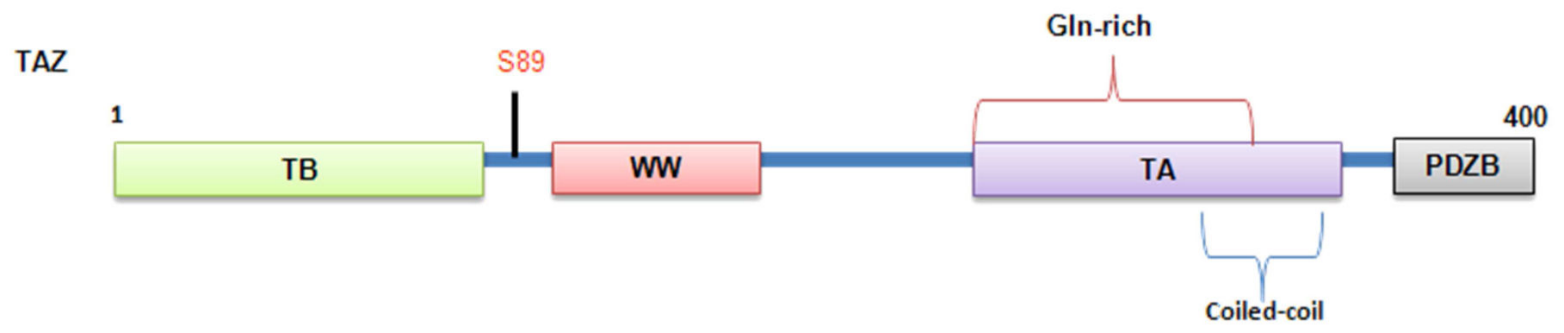
TAZ structure domain TB: TEAD binding domain; WW: WW domain; TA: Transactivation domain, which contains a Gln-rich region (194-241 aa) and Coiled-coil region (225-259 aa); PDZB: PDZ-binding domain; S89-LATS phosphorylation site.

## Protein

### Description

TAZ is a downstream transcriptional coactivator in the Hippo pathway (Kanai et al., 2000; Lei et al., 2008; Hong and Guan, 2012). TAZ has one WW domain which allows its interaction with PPxY motif-containing proteins such as LATS kinases in the Hippo pathway as well as other transcription factors (TFs). Its N-termini contains a Tead-binding (TB) domain, through which TAZ can bind to TEAD, which is a well-known TF involved in cell proliferation and anti-apoptosis. In its C-termini, there is a Transcriptional Activation (TA) domain which contains a Gln-rich region (amino acid (aa.) 194-241) and Coiled-coil region (aa. 225-259). From 394 to aa. 400 of TAZ, there is a PDZ-binding domain, which has been found important for transcriptional coactivating function of TAZ (Wang et al., 2009; Liu et al., 2011).

### Expression

TAZ is expressed in various tissues, and high expression of TAZ has been found in thyroid, kidney, heart, placenta and lung.

### Localisation

TAZ localizes in both cytoplasm and nucleus. Normally, in the nucleus, TAZ can possess its transcription-activating function and help initiate target genes’ expressions through binding with related transcriptional factors. And the localization of TAZ can be regulated by cell-cell contact. Once cells get confluent (high cell-cell contact), the Hippo pathway will be activated (Zhao et al., 2011). As a result, TAZ will be phosphorylated on S89, initiating its binding with 14-3-3 (Lei et al., 2008; Zhao et al., 2008), which will anchor TAZ in the cytoplasm. Besides, the interaction with some proteins, such as AMOT and ZO-1, can also localize TAZ to cell membrane (Chan et al., 2011; Remue et al., 2010).

### Function

TAZ functions as an oncogene. Over-expression of TAZ induces increased cell proliferation, epithelial-mesenchymal transition (EMT), cell migration and transformation (Chan et al., 2009; Lai et al., 2011). In addition, enhanced levels of TAZ causes drug resistance by activating CTGF and Cyr61 (Lai et al., 2011).

TAZ is also involved in mesenchymal stem cell differentiation. TAZ can activate TF RUNX2 to induce osteoblast differentiation, while TAZ binds and inhibits PPARG TF, which further blocks adipocyte differentiation. Besides, TAZ also regulates myoblast differentiation by enhancing TF MyoD-dependent myogenic gene expression (Hong et al., 2005; Jung et al., 2009; Cho HH et al., 2010).

TAZ relates to tissue homeostasis and development as well. TAZ knockout mice develop Polycystic Kidney Disease (PKD) and emphysema, suggesting an important role of TAZ in renal and lung development (Liu et al., 2011).

TAZ also plays a role in mechanotransduction. Extracellular matrix stiffness or confined adhesiveness can cause TAZ retention in nuclear, which, therefore contributes to cell proliferation, mesenchymal stem cell differentiation as well as cancer malignant progression (Dupont et al., 2011).

### Homology

TAZ gene is conserved across species. Homologous proteins have been found in chimpanzee, dog, cow, mouse, rate, chicken and zebrafish.

## Mutations

### 

#### Note

TAZ has a missense mutation (F299V), which was detected at 7% and 10% in primary mammary tumor and xenograft respectively, as well as 28% mutant allele frequency in metastatic breast cancers (Ding et al., 2010).

## Implicated in

### Non-small cell lung cancer

#### Note

High level of TAZ has been found in different non-small cell lung cancer (NSCLC) cell lines.

TAZ overexpression in immortalized non-tumorigenic lung epithelial cells causes increased cell proliferation and transformation, whereas TAZ knockdown in NSCLC cells significantly reduces tumor cell proliferation and tumor growth in nude mice (Zhou et al., 2011).

Significantly, TAZ expression was found associated with lung adenocarcinoma, metastasis, poorer differentiation and poor prognosis (Xie et al., 2012). Lung cancer patients with negative TAZ expression have prolonged overall survival (Lau et al., 2014).

### Colorectal cancer

#### Note

High levels of TAZ mRNA are significantly correlated with shorter survival in colorectal cancer patients. This was due to the increased levels of TAZ downstream target genes CTGF and AXL, which are involved in colorectal cancer development (Yuen et al., 2013).

### Breast cancer

#### Note

TAZ has been found correlated with breast cancers. The breast cancer cell lines have high expression of TAZ and 20% of breast cancer samples have TAZ overexpression (Chan et al., 2009). TAZ causes increased cell migration through activation of BMP4, and resistance to chemotherapeutic drug Taxol through downstream Cyr61 and CTGF (Lai et al., 2011; Lai and Yang, 2013). TAZ can also cause increased cell proliferation and tumorigenesis by activating KLF5 through inhibition of KLF5 degradation (Zhao et al., 2012). Also, TAZ has been suggested to play a role in breast cancer stem cell self-renewal and tumor-initiation capabilities (Cordenonsi et al., 2011; Bartucci et al., 2014). Moreover, TAZ is also found amplified in 44% basal-like and 30% luminal breast cancer (Skibinski et al., 2014).

### Tongue squamous cell carcinoma (TSCC)

#### Note

TSCC cells and specimens have significantly higher expression of TAZ than those in non-cancerous cells and normal tongue mucosa. Overexpression of TAZ in TSCC was significantly associated with tumor size, clinical stage and reduced overall and disease-free survival (Wei et al., 2013).

### Polycystic kidney disease (PKD)

#### Note

TAZ knockout mice develop PKD during development. NEK1 kinase can phosphorylate TAZ, which can disable TAZ’s role in promoting the degradation of PC2, a protein involved in ciliogenesis. The proper balance of NEK1 and TAZ will help keep a good level of PC2, which will protect kidney from PKD (Tian et al., 2007; Yim et al., 2011).

### Holt-Oram syndrome

#### Note

TAZ can interact with and activate transcription factor TBX5, which is essential in cardiac and limb development. In Holt-Oram syndrome, TBX5 has a truncated mutation, which will lose its interaction with TAZ and therefore, fail to activate genes involved in cardiac and limb development (Murakami et al., 2005).
